# Astrocyte Ca^2+^ Waves and Subsequent Non-Synchronized Ca^2+^ Oscillations Coincide with Arteriole Diameter Changes in Response to Spreading Depolarization

**DOI:** 10.3390/ijms22073442

**Published:** 2021-03-26

**Authors:** Réka Tóth, Attila E. Farkas, István A. Krizbai, Péter Makra, Ferenc Bari, Eszter Farkas, Ákos Menyhárt

**Affiliations:** 1 Department of Medical Physics and Informatics, Faculty of Medicine and Faculty of Science and Informatics, University of Szeged, Korányi fasor 9, 6720 Szeged, Hungary; tothreka@outlook.com (R.T.); makra.peter@med.u-szeged.hu (P.M.); bari.ferenc@med.u-szeged.hu (F.B.); 2 Neurovascular Unit Research Group, Molecular Neurobiology Research Unit, Institute of Biophysics, Biological Research Centre, Eötvös Loránd Research Network (ELKH), Temesvári krt 62, 6726 Szeged, Hungary; farkas.attilae@brc.hu (A.E.F.); krizbai.istvan@brc.hu (I.A.K.); 3 Institute of Life Sciences, Vasile Goldis Western University, Revolutiei Blvd no. 94, 310025 Arad, Romania

**Keywords:** arteriole, astrocyte, Ca^2+^ oscillation, cerebrovascular, spreading depolarization

## Abstract

Spreading depolarization (SD) is a wave of mass depolarization that causes profound perfusion changes in acute cerebrovascular diseases. Although the astrocyte response is secondary to the neuronal depolarization with SD, it remains to be explored how glial activity is altered after the passage of SD. Here, we describe post-SD high frequency astrocyte Ca^2+^ oscillations in the mouse somatosensory cortex. The intracellular Ca^2+^ changes of SR101 labeled astrocytes and the SD-related arteriole diameter variations were simultaneously visualized by multiphoton microscopy in anesthetized mice. Post-SD astrocyte Ca^2+^ oscillations were identified as Ca^2+^ events non-synchronized among astrocytes in the field of view. Ca^2+^ oscillations occurred minutes after the Ca^2+^ wave of SD. Furthermore, fewer astrocytes were involved in Ca^2+^ oscillations at a given time, compared to Ca^2+^ waves, engaging all astrocytes in the field of view simultaneously. Finally, our data confirm that astrocyte Ca^2+^ waves coincide with arteriolar constriction, while post-SD Ca^2+^ oscillations occur with the peak of the SD-related vasodilation. This is the first in vivo study to present the post-SD astrocyte Ca^2+^ oscillations. Our results provide novel insight into the spatio-temporal correlation between glial reactivity and cerebral arteriole diameter changes behind the SD wavefront.

## 1. Introduction

The outcome of acute brain injury worsens with the occurrence of secondary pathophysiological events that compromise cerebral perfusion in the subacute and chronic phase of injury. Notably, spreading depolarization (SD) waves [1,2], which generate in metabolically instable or metastable hot zones of the nervous tissue [3], are coupled with a typical cerebral blood flow (CBF) response that aggravates the local metabolic crisis [4,5]. Of the subsequent elements of the SD-coupled CBF response (i.e., initial, brief hypoperfusion; dominant, peak and late hyperemia; concluding, long-lasting oligemia), the initial transient hypoperfusion gains ground in sub-optimally perfused tissue by becoming elongated or persistent at the expense of hyperemia [6,7]. This phenomenon, which is known as spreading ischemia [5,8], impedes the replenishment of ATP stores, sustains SD, and ultimately contributes to the evolution of secondary injury [9].

Astrocyte Ca^2+^ waves have been considered as reliable indicators of astrocyte activation implicated in the glutamate release from Schaffer collateral terminals that innervate CA1 pyramidal neurons [10,11]. Additionally, astroglial cells directly potentiate neuronal excitability by Ca^2+^ dependent gliotransmission, a process during which the astrocyte derived glutamate evokes slow inward currents (SICs) in pyramidal neurons to support their synaptic synchronization [10]. Furthermore, astrocytic Ca^2+^ transients contribute to the regulation of cerebrovascular tone [12,13]. Importantly, astrocyte Ca^2+^ waves have been linked to both cerebrovascular dilation during neurovascular coupling [14,15], or to vasoconstriction after acute, injurious cerebrovascular events [16,17]. This bipolar regulation of the vascular tone is possibly dependent on tissue oxygenation and metabolic state [18], or the amplitude of the Ca^2+^ transients and resting Ca^2+^ levels [17,19]. As in physiological neurovascular coupling [20,21], astrocytes are thought to release vasoactive substances (e.g., arachidonic acid and its prostanoid derivatives) during SD, which cause the contraction or relaxation of cerebrovascular smooth muscle cells [4,22,23,24,25]. In accordance with these results, SD was found to trigger fast astrocyte Ca^2+^ waves that temporally coincided with arteriolar constrictions in mice [26,27,28,29]. Finally, we have previously reported that the SD-related arteriole constriction is driven by perivascular K^+^ release through large-conductance Ca^2+^-activated potassium channels (BK channels) on astrocyte endfeet [24].

In this study, we set out to explore the spatio-temporal relationship between the astrocytic Ca^2+^ events and the arteriolar diameter variations during SD. We describe a characteristic pattern of large frequency astrocyte Ca^2+^ oscillations emerging a couple of minutes behind the Ca^2+^ wave of SD. The oscillations were reminiscent of a series of repetitive astrocytic Ca^2+^ spikes, which have recently been described in live brain slice preparations [30]. Here, we provide evidence for the first time that the post-SD astrocyte Ca^2+^ oscillations occur in vivo, in the normally perfused, intact brain of anesthetized mice. We examined the characteristic pattern of these Ca^2+^ oscillations and their spatio-temporal coincidence with SD-related cerebrovascular tone adjustments.

## 2. Results

### 2.1. Non-Synchronized Astrocyte Ca^2+^ Oscillations Accompany the Concurrent Ca^2+^ Wave of Spreading Depolarization in the Mouse Somatosensory Cortex

Here, we set out to explore the spatio-temporal relationship between the astrocytic Ca^2+^ changes and the arteriolar diameter variations during SD. We followed the design of our previously reported anesthetized mouse model, in which SDs were evoked by topical 1 M KCl application [24]. SD occurrence was confirmed by (i) the typical 77.5 ± 25.2 µm/s propagation velocity of the astrocyte Ca^2+^ wave [28], and (ii) the characteristic cerebrocortical microvascular changes associated with SD [24].

**Figure 1:**
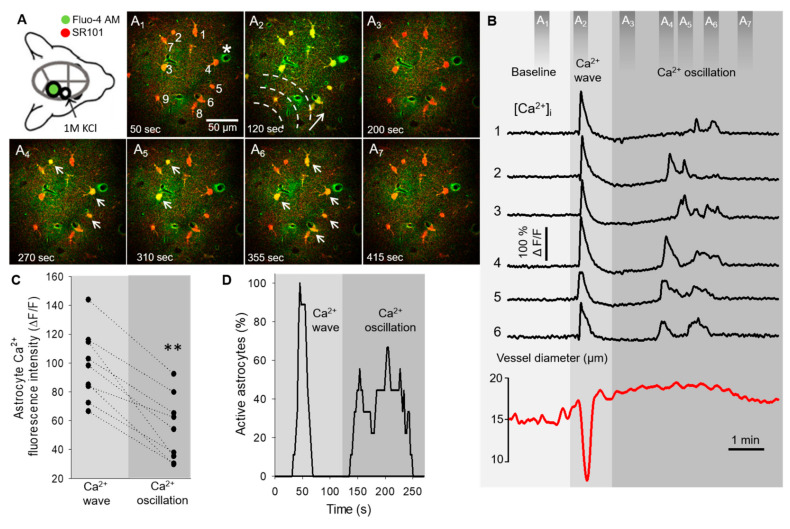
Astrocyte Ca^2+^ dynamics during spreading depolarization (SD) in the mouse somatosensory cortex. (**A**) Schematic illustration of the closed cranial window preparation indicates the position of the imaging site (green). SD events were triggered by topical application of KCl in a smaller rostral open craniotomy (open circle). Images (**A**_1–7_) demonstrate astrocyte Ca^2+^ changes (Fluo-4 AM, green) associated with SD. Astrocytes (numbered 1–6 on **A**_1_) were selectively labeled by SR101 (red). Dashed lines and arrow (**A**_2_) denote the direction of SD propagation; white arrows are pointing at astrocyte somata displaying Ca^2+^ oscillations (**A**_4–6_). (**B**) Astrocyte Ca^2+^ changes (i.e., wave and oscillations) extracted from regions of interests (numbered 1–6 in **A**_1_) coincide with arteriole diameter changes (labeled with * in **A**_1_) during SD. Dark grey bars (top) indicate time points of the corresponding images (**A**_1–7_). (**C**) Ladder plot shows the peak fluorescence maximum (ΔF/F) of Ca^2+^ waves and subsequent oscillations derived from nine cells. (**D**) Percentage of astrocytes (*n* = 9 in total) displaying Ca^2+^ changes at each time point during acquisition. Ca^2+^ events were defined as ΔF/F ≥ 13% with respect to baseline fluorescence. Images were taken at a cortical depth of 55–75 μm. Data are given as mean ± st.dev. Two-tailed paired *t*-test was used for statistical analysis with the level of significance set at *p* ** < 0.01.

**Figure 2:**
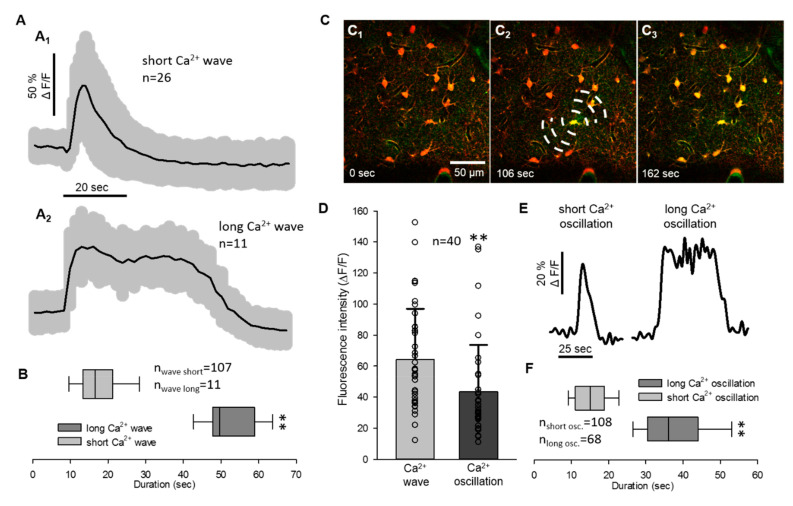
Quantitative analysis of the spreading depolarization related astrocyte Ca^2+^ waves and oscillations. (**A**) Traces of SD related short and long Ca^2+^ waves (mean ± st.dev.). (**B**) Duration of SD related Ca^2+^ waves. (**C**) Images represent Ca^2+^ changes (Fluo-4 AM, green) of astrocytes (SR101, red) with the propagation of SD. Dashed lines in **D**_2_ indicate the origin of SD and direction of propagation. Note that images (**C**_1_–**C**_3_) represent a long Ca^2+^ wave with a focal SD origin in the field of view. (**D**) Peak fluorescence maxima (ΔF/F) of astrocyte Ca^2+^ waves and oscillations derived pairwise from 40 cells. (**E**) Representative traces of short and long Ca^2+^ oscillations after SD. (**F**) Duration of the SD related Ca^2+^ oscillations. Images were taken at a cortical depth of 55–75 μm. Data are given as mean ± st.dev. Statistical analysis relied on a Mann-Whitney Rank Sum Test (**B**,**F**) and two-tailed paired T test (**D**); *p* < 0.01 **.

The typical intracellular fast astrocyte Ca^2+^ changes and the coincident variation of arteriolar diameter, both associated with SD, are represented in the network of six astrocytes in Figure 1. Due to the fast propagation speed of the SD-related Ca^2+^ wave (77.5 ± 25.2 µm/s), the peak fluorescence maximum was temporally synchronized in cells of the field of view (Figure 1A_2,_B). The Ca^2+^ wave was also temporally coincident with the SD-related arteriolar constriction (Figure 1A_2_,B, Supplementary Video S1). In contrast, at a 183.64 ± 89.21 s delay with respect to the synchronized Ca^2+^ wave, the same astrocyte network showed non-synchronized Ca^2+^ oscillations (Figure 1A_4-6_, Supplementary Video S1). These oscillations were of three times higher frequency (0.66 ± 0.32 event/minute/cell) when compared to spontaneous basal Ca^2+^ activity (0.19 ± 0.032 event/minute/cell). The oscillations in individual cells were random and repetitive. Temporal coincidence of the Ca^2+^ spikes between cells were not observed, but the typical pattern of Ca^2+^ oscillations occurred in association with the plateau of the SD-related arteriolar dilation (Figure 1B, Supplementary Video S1). The peak fluorescence maximum of Ca^2+^ oscillations was smaller compared to the maximum amplitude of the prior Ca^2+^ wave in the same cells (54.02 ± 22.65 vs. 98.06 ± 24.35 ΔF/F, Ca^2+^ oscillation vs. Ca^2+^ wave) (Figure 1C and Figure 2D). Additionally, fewer astrocytes were involved in Ca^2+^ oscillations at a given time point, in contrast with Ca^2+^ waves, which engaged all astrocytes in the field of view virtually simultaneously (Figure 1D).

As the duration of Ca^2+^ waves and oscillations each displayed two distinct, non-overlapping pools of data, both the Ca^2+^ waves and the oscillations were divided into two subgroups by their temporal dynamics (Figure 2A,E). The astrocyte Ca^2+^ wave was short (≤33 s) in most of the cases (five out of six animals), when SD travelled with a wave front from the origin of the rostral craniotomy to the imaging field (Figure 2A1). However, in one recording, the Ca^2+^ wave of SD appeared to emerge from a single astrocyte and spread to neighboring somata (Figure 2C_1–3_). The Ca^2+^ waves measured in individual astrocyte somata in this recording were significantly longer lasting (51.85 ± 7.07 vs. 18.2 ± 8.77 s; long Ca^2+^ wave vs. short Ca^2+^ wave; Figure 2A2,B). The longer duration of the Ca^2+^ wave (>33 s) cannot be attributed to the image sampling artifact, since the second SD in the same recording was associated with the short Ca^2+^ wave (14.94 ± 4.94 s).

The duration of Ca^2+^ oscillations was not linked to the direction of SD origin, because both short (<25 s) and long (>25 s) Ca^2+^ oscillations were observed with both propagation patterns observed (38.23 ± 10.36 vs. 14.94 ± 4.94 s: long Ca^2+^ osc. vs. short Ca^2+^ osc., Figure 2E,F). Peak fluorescence intensity of Ca^2+^ waves and oscillations extracted from all recordings displayed the same distribution as presented previously in Figure 1D (64.35 ± 32.62 vs. 43.5 ± 30.15 ΔF/F, Ca^2+^ wave vs. Ca^2+^ oscillation).

### 2.2. Astrocyte Ca^2+^ Waves Coincide with Arteriolar Constrictions While Ca^2+^ Oscillations Occur during Vasodilation

**Figure 3:**
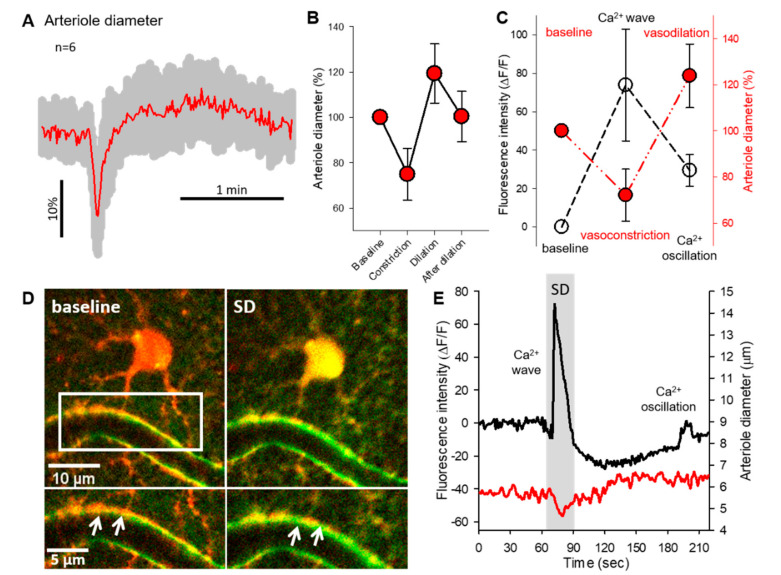
Characterization of arteriole diameter changes associated with astrocyte Ca^2+^ events. (**A**) Normalized vessel diameter changes (mean ± st.dev.) associated with SD. (**B**) Absolute and relative variations of arteriolar diameter coupled to SDs. (**C**) Concurrent variations of arteriole diameter (expressed in %) and astrocytic Ca^2+^ peak fluorescence (ΔF/F). (**D**) Representative images demonstrate a cortical astrocyte (red) opposed to the arteriole wall at the baseline condition and during SD. Green fluorescent signal increase labels the Ca^2+^ wave of SD. Inserts mark the astrocyte endfeet-vessel wall connection (**D**, bottom). White arrowheads depict the increased green, fluorescent signal at the endfoot-arteriolar surface during SD. (**E**) Representative traces of the SD coupled Ca^2+^ wave, oscillation (black) and arteriolar response (red). Traces were extracted from the astrocyte soma and nearby arteriole shown in Panel **D**. Data are given as mean ± st.dev.

Intracortical penetrating arterioles showed biphasic diameter changes during SDs (Figure 3A). The vessel response consisted of a transient vasoconstriction (74.89 ± 11.46% of baseline) that was followed by a subsequent dilation (119.34 ± 13.09% of baseline) (Figure 3A,B). After the dilation phase, all arterioles recovered to the baseline diameter (100.39 ± 11.07%, Figure 3B). The arteriole diameter changes during SDs were spatio-temporally coincident with glial Ca^2+^ events. The SD-related Ca^2+^ wave peak fluorescence maximum was associated with the maximum degree of vasoconstriction (Ca^2+^ wave: 73.82 ± 29.1 ΔF/F; diameter change: 72.07 ± 11.31% of baseline). In contrast, the smaller amplitude Ca^2+^ oscillation peaks occurred during the highest degree of vasodilation (Ca^2+^ oscillation: 29.04 ± 8.29 ΔF/F; diameter change: 123.89 ± 13.8% of baseline) (Figure 3C). Although the imaging resolution did not allow the consistent discrimination of Ca^2+^ fluctuations in perivascular astrocyte endfeet, representative images in Figure 3D demonstrate a single astrocyte with a perivascular endfoot during the Ca^2+^ wave of SD (Figure 3D). At the temporal resolution used, the somatic Ca^2+^ wave appeared synchronous in time with perivascular endfeet Ca^2+^ increase (Figure 3D inserts). The same cell displayed the Ca^2+^ wave coincident with the rapid vasoconstriction, and one Ca^2+^ oscillation spike during the arteriole dilation (Figure 3E).

## 3. Discussion

In the present study, we visualized astrocyte Ca^2+^ changes and vascular responses associated with SD in the anesthetized mouse somatosensory cortex. This is the first in vivo study to describe the delayed astrocyte Ca^2+^ oscillations following SD, and to investigate their temporal relationship to arteriolar diameter changes.

Astrocyte Ca^2+^ waves indicate glial activity in response to mechanical, electrical, or pharmacological stimulation, which propagate from cell-to-cell with a relatively slow velocity of 10–30 µm/s [31,32,33]. Under pathological conditions like seizures or SD, the propagation velocity within the astrocyte network increase, and astrocytes display much faster Ca^2+^ waves (50-80 µm/s) [28,29]. The Ca^2+^ waves measured in our model were typical of SD because they propagated at a speed of 77.5 ± 25.2 µm/s, delineating the direction of SD propagation (Figure 1A-D). Additionally, the arteriolar constrictions coupled with the Ca^2+^ waves stood in good agreement with earlier reports, and confirmed SD occurrence in our model [28].

Examining the duration of the SD related Ca^2+^ events in individual astrocytes, we discriminated short (≤33 s) and long (>33 s) astrocyte Ca^2+^ waves during SD. A long Ca^2+^ wave was captured in one experiment, in which the SD-related Ca^2+^ elevation spatially radiated from a single astrocyte (Figure 2C_1–3_).

We confirmed the occurrence of delayed random Ca^2+^ oscillations in astrocytes 2–4 min after the passage of the SD-related Ca^2+^ wave, occurring at a frequency of 0.66 ± 0.32 events/min/cell (0.01 Hz) in a chosen astrocyte soma. In agreement with the slice results of Wu et al., we observed that the frequency of these post-SD oscillations was three times higher than the spontaneous Ca^2+^ fluctuation of astrocytes during baseline (0.19 event/min/cell) [30]. Although the propagation of these spontaneous Ca^2+^ oscillations in cell cultures and brain slices was shown in an earlier study [27,34], we did not discern synchronization of the Ca^2+^ oscillations among the cells [34]. The cell-to-cell propagation of the oscillations could have escaped detection in our preparations in case the astrocyte linked to the oscillating cell fell out of the plane of view, or if the velocity of cell-to-cell propagation of the signal was too rapid with respect to the recording’s sampling frequency. Since post-SD Ca^2+^ oscillations have been shown to promote slow inward currents (SICs) in neurons [30,34] the increased Ca^2+^ activity of astrocytes might contribute to the NMDA receptor driven hyperexcitability after SD [35]. Astrocyte Ca^2+^ oscillations had a smaller fluorescent peak amplitude when compared to the prior Ca^2+^ waves, which could be due to different mechanisms in the background of these two events. For instance, antagonism of GABA_B_ receptors, depletion of intracellular Ca^2+^ stores, or blockade of IP3 receptors all attenuated the Ca^2+^ oscillations, but had no significant effect on the SD-related Ca^2+^ wave [30,34]. One could therefore speculate that while Ca^2+^ waves rely on multiple pathways mobilizing external Ca^2+^, post-SD oscillations are largely dependent on Ca^2+^ release from the endoplasmic reticulum (ER) or mitochondria.

The SD-linked arteriolar diameter changes consisted of a short constriction and a subsequent dilation, confirming our earlier findings [24]. The spatial and temporal coincidence of the astrocyte Ca^2+^ wave and the arteriolar constriction in our present study also stands in good agreement with previous reports [28,29]. The SD-linked arteriole constriction was proposed to be mediated by either astrocytic Ca^2+^ dependent arachidonic acid release in brain slice preparations [36], or perivascular K^+^ release through BK channels with KCl evoked SDs in anesthetized mice [24]. Such mechanistic association was not observed between the random astrocyte Ca^2+^ oscillations and arteriolar dilation after SD.

The causality between the post-SD Ca^2+^ oscillations and the vasodilation element of the vascular response is rather unlikely, because the onset of vasodilation preceded the Ca^2+^ oscillations, which clearly emerged with the peak of the arteriolar dilation. Furthermore, it has also been shown that SD-associated glial Ca^2+^ transients (either short and relatively small, or prolonged and large) do not necessarily coincide with arteriole diameter changes [28]. Therefore, we propose a negligible role of post-SD oscillations in the SD-caused arteriole dilation.

Taken together, our results provide the first in vivo characterization of delayed astrocyte Ca^2+^ oscillations following SD, and are complementary to the in vitro findings of Wu et al. [30]. Although SD is primarily a neuronal depolarization wave, the role of astrocytes in the electrophysiological restoration of the nervous tissue after SD, and in ischemia, is increasingly more recognized [37]. Therefore, better understanding of astroglial biology is expected to improve our understanding of SD and its consequences and may offer more specific therapeutic targets to treat acute cerebrovascular disorders in which SD is relevant.

## 4. Materials and Methods

### 4.1. Animals

The experimental procedures were approved by the National Food Chain Safety and Animal Health Directorate of Csongrád County, Hungary. The procedures were performed according to the guidelines of the Scientific Committee of Animal Experimentation of the Hungarian Academy of Sciences (updated Law and Regulations on Animal Protection: 40/2013. (II. 14.) Gov. of Hungary), following the EU Directive 2010/63/EU on the protection. The experiments were reported in compliance with the ARRIVE guidelines.

Male C57BL/6 mice (8–10 weeks old, *n* = 6) were anesthetized with 1% Avertin (20 µL/g, i.p.), and mounted on a stereotactic frame incorporating a heating pad [24]. A cranial window (d = 3 mm) was prepared on the right parietal bone, and the dura was retracted. For astrocyte intracellular calcium imaging, the exposed brain surface was first loaded topically with a green, fluorescent calcium indicator Fluo 4-AM (45 µM in aCSF, Thermo Fisher, Waltham, MA, USA) and incubated for 15 min. Subsequently, to label astrocytes, the red fluorescent dye sulforhodamine 101 (SR101, 80 µM in aCSF, Thermo Fisher, Waltham, MA, USA) [38,39,40], was applied topically, and left on the brain surface for a further 15 min. A perceived limitation of our SR101 labeling protocol may be the potential non-specific cellular uptake of the dye [41]. We identified cells selected for the analysis as astrocytes on the basis of their morphology (e.g., ramified structure, endfoot processes embracing vessels) in the superficial layers of the cortex, which is particularly rich in astrocytes due to the formation of the superficial glial limiting membrane. Although the labeling of oligodendorcytes cannot be excluded, oligodendrocytes display round somata, and the superficial layer of the cortex examined here has been known to be largely devoid of oligodendrocytes [42]. The predominant uptake of the dye by astrocytes, in contrast with neurons, was also substantiated by the obvious, dark shadow of neuronal cell bodies in the neuropil in the preparations. The craniotomy was then closed with a microscopic cover glass. A second, smaller trepanation was drilled rostral to the first craniotomy, to be used for SD elicitation. A glass capillary connected to a syringe pump (CMA/100, CMA/Microdialysis, Solna, Sweden) was filled with 1 M KCl, and was fastened to the skull with acrylic dental cement, with its tip positioned at the cortical surface within the rostral trepanation.

### 4.2. Multiphoton Microscopy

Multiphoton excitation was performed at the 810 nm wavelength according to protocols described previously [24]. In vivo intracranial microscopy was performed with a FEMTO 3D Dual microscope (Femtonics Ltd., Budapest, Hungary) using a 20× large working distance water objective (XLUMPLFLN-20XW, Olympus, Tokyo, Japan) and MES software (v4.6.2336, Femtonics, Budapest, Hungary). Two-photon excitation was performed with a Mai Tai HP Ti-sapphire laser (RK TECH Ltd., Budapest, Hungary) at 810 nm, which was found optimal for Fluo-4 AM excitation, and adequate for SR101. Emission was detected with gallium arsenide phosphide photomultipliers, equipped with the appropriate color filters. Laser power was set to 10–40%, depending on the depth of imaging (0–300 μm from the brain surface), photomultiplier voltages were set to 70%. Final imaging depth in the somatosensory cortex was 55–85 µm, where a z-stack with 5 μm vertical steps was recorded at the area of interest for the identification of astrocytes. Image sequences were taken of the desired cells at approximately 1 μm/pixel spatial and 0.8–2.5 Hz temporal resolution. After acquiring baseline images, SD was triggered repeatedly at intervals of 15–20 min in the rostral cranial window by the ejection of 1–3 μL 1 M KCl to the brain surface through the glass capillary. SD evolution was confirmed by the occurrence of synchronous, propagating astrocytic calcium waves (Fluo 4-AM intensity increase, green channel) and the associated changes in arteriole diameter.

### 4.3. Data Analysis

Multiphoton image stacks were processed offline. Image stacks were auto leveled, background subtracted, and converted to RGB color in Fiji. Movement artifacts were corrected using the “Template Matching” plugin in Fiji. The SD associated and subsequent intracellular calcium changes were measured on green fluorescent images (ΔF/F) by placing 4–10 µm ROIs on the soma of selected astrocytes. Cells expressing both Ca^2+^ waves and subsequent Ca^2+^ oscillations were considered for comprehensive analysis (Figure 1). The vessel identification protocol followed previously established principles [24]. In brief, arterioles were identified by their pial latero-medial anatomical branching. Arterioles and venules were differentiated with the help of 3D reconstruction relying on a z-stack of two-photon images, as previously described [24]. The analyzed penetrating arterioles and first order arterioles were direct branches of the pial arterioles. In addition, venules could be reliably discriminated from arterioles on the basis of their irresponsiveness to SD [24]. Although we did not perform vascular lumen labeling, the non-specific accumulation of green Fluo-4 AM dye in vascular smooth muscle cells and/or perivascular spaces guided the exact evaluation of vascular diameter changes (Supplementary Video S1). Additional, specific criteria to include a vessel into the analysis were as follows: (i) The intracellular Ca^2+^ wave of SD must have propagated fully over the astrocytes next to the vessel; (ii) The baseline diameter of penetrating arterioles was above 5 μm in order to have reliable assessment of vasoconstriction with respect to pixel size (i.e., 1 μm); and (iii) The penetrating arteriole optimally appeared in cross sectional view. Vascular diameters were either measured manually at baseline (i.e., prior to SD), maximum constriction, subsequent maximum dilation, and recovery or using the” Diameter” plugin in Fiji [43]. Recordings formed the subject of analysis in case vascular diameter alterations occurred in the presence of astrocyte Ca^2+^ waves (Figure 3C).

Quantitative data are given as mean ± standard deviation (st.dev.). Statistical analysis was conducted with the software SigmaPlot 12.5 (Systat Software, Inc., San Jose, CA, USA). Datasets were evaluated by a two-tailed paired T-test or a Mann-Whitney Rank Sum Test. Levels of significance were set at *p* < 0.05* or *p* < 0.01**. Distinct statistical methods are provided in each figure legend in detail.

## Data Availability

The datasets used and/or analyzed during the current study are available from the corresponding authors on reasonable request.

## Supplementary Materials

The following are available online at https://www.mdpi.com/article/10.3390/ijms22073442/s1, Video S1: manuscript-supplementary.avi.
